# Excessive Dynamic Airway Collapse: An Unexpected Contributor to Respiratory Failure in a Surgical Patient

**DOI:** 10.1155/2015/596857

**Published:** 2015-06-08

**Authors:** Michael R. Lyaker, Victor R. Davila, Thomas J. Papadimos

**Affiliations:** Department of Anesthesiology, The Ohio State University Wexner Medical Center, 410 W. 10th Avenue, Columbus, OH 43210, USA

## Abstract

Central airway collapse plays a significant, underrecognized role in respiratory failure after extubation of critically ill patients. Historically, airway collapse has been attributed to tracheomalacia (TM), softening of the cartilage in the trachea and other large airways. More recently, excessive dynamic airway collapse (EDAC) has been described as a distinct process unrelated to a loss of cartilaginous airway support. EDAC is caused by the posterior wall of the trachea bulging forward and causing airway obstruction during exhalation. This process is exaggerated when intrathoracic pressure is increased and results in a clinical picture of coughing, difficulty clearing secretions, dyspnea, and stridor. The increased use of computerized tomography and fiberoptic bronchoscopy has identified varying degrees of EDAC and TM in both symptomatic and asymptomatic individuals. This has led to renewed consideration of airway collapse and the different processes that contribute to it. Here we describe a 43-year-old morbidly obese patient who failed repeated attempts at extubation after elective hysterectomy. We will discuss the processes of EDAC and TM, describe how this condition contributed to this patient's respiratory failure, and review diagnosis and management options.

## 1. Introduction

During normal respiration the cross-sectional area of the intrathoracic trachea increases in response to the negative pleural pressure generated by inspiration and decreases during exhalation [1, 2]. The degree of change in the anterior-posterior diameter can vary widely [3]. Any process that further elevates intrathoracic pressure, such as coughing and the Valsalva maneuver, can exacerbate collapse thereby increasing expiratory airway obstruction [2]. Pathologic airway collapse presents as stridor, wheezing, cough, expiratory dyspnea, or difficulty clearing secretions [4]. Often these findings lead to the misdiagnosis of asthma or chronic obstructive pulmonary disease (COPD) [5].

Historically, the terms tracheomalacia (TM) and “tracheal softening” (or tracheobronchomalacia when it involves the bronchi) have been used to describe any hyperdynamic or pathologic collapse of the upper airways [1, 4]. This process can be either congenital, as in the pediatric population, or acquired due to long-standing infection, COPD, trauma, tracheostomy, chronic tracheal inflammation (e.g., from polychondritis), or external compression [1, 2, 4]. However, in recent decades, bronchoscopy and the use of newer diagnostic modalities such as dynamic computerized tomography (CT) have increased recognition that airway collapse is a common, underrecognized, and heterogeneous process [2, 3, 6]. This awareness has led some authors to draw a distinction between “true” TM, which results from cartilaginous softening, and expiratory obstruction caused by anterior bowing of the posterior tracheal wall [7, 8]. The later process has been termed excessive dynamic airway collapse (EDAC) [7, 8]. It is important to note that there is no clear consensus on how to determine the clinical significance of these findings in individuals [3] or when and how such patients should be treated [1, 4, 8, 9].

This case report discusses a patient who presented with significant unrecognized EDAC after elective hysterectomy. Challenges to extubation related to morbid obesity and postoperative reduction in functional residual capacity (FRC) were anticipated. Nonetheless, there were repeated unexpected failures to liberate her from mechanical ventilation after surgery. CT scan and bronchoscopy led to the diagnosis of EDAC and the determination that this condition was a significant barrier to successful extubation.

## 2. Case Presentation

A forty-three-year-old morbidly obese female with a history of hypertension, treated with lisinopril, presented to her gynecologist complaining of vaginal bleeding. Her workup included an endometrial biopsy which revealed grade I endometrial adenocarcinoma. In preparation for hysterectomy, she was sent to a preoperative clinic staffed by our anesthesiology department. She presented with a BMI of 62 (height 69 inches, weight 190 kg), poor functional status, anemia (hemoglobin 9.0), and multiple risk factors for obstructive sleep apnea. No other comorbidities were identified during this evaluation. A preoperative echocardiogram did not suggest elevated pulmonary artery pressures, valvular disease, or dysfunction of either ventricle.

The patient successfully underwent transabdominal hysterectomy, resection of an abdominopelvic mass, and right-sided salpingo-oophorectomy. Although the procedure was initially attempted robotically, it was converted to an open approach due to technical factors. To facilitate adequate exposure, a midline laparotomy incision was performed from just above the pubis to 8 cm above the umbilicus. The rest of the three-hour surgery proceeded without incident. Blood loss was estimated at 400 mL, urine output was 100 mL, and a total of 3.1 liters of crystalloid was administered. At the end of the procedure neuromuscular blockade was reversed; the patient was placed in a semirecumbent position and extubated when fully awake. Soon after extubation the patient's breathing became rapid and labored. In spite of the administration of a high concentration of oxygen by face mask, her oxygen saturation decreased to 85%. The endotracheal tube was reinserted and she was sent to the surgical intensive care unit (SICU).

The patient was mechanically ventilated using volume control ventilation (set rate of 12, tidal volume (TV) of 550 mL, positive end-expiratory pressure (PEEP) of 8 cm H_2_O, and peak inspiratory pressure (PIP) of 25 cm H_2_O). Her FiO_2_ was weaned from 100% to 50% over the first few hours. Her total respiratory rate ranged from 22 to 28 breaths per minute (BPM). She was fully awake and frequently anxious. The postoperative chest X-ray demonstrated low lung volumes. A single unit of red blood cells was administered for hemoglobin of 6.8 g/dL. The following morning a spontaneous breathing trial (SBT) was attempted by decreasing the patient's pressure support to 0 cm of H_2_O while keeping PEEP at 5 cm H_2_O. Within several minutes the patient became severely tachypneic and dyspneic. She was converted to volume support ventilation using a goal TV of 450 mL (6.4 mL/kg ideal body weight), requiring PIP of 23 cm H_2_O.

On postoperative day (POD) 2, the patient aspirated during her SBT and her oxygen requirement increased to 80%. Her trachea was thoroughly suctioned, the PEEP was increased to 10 cm H_2_O, and she was placed on assist control ventilation with a TV of 450 mL and rate of 12 BPM. The inspiratory time was also increased to improve oxygenation. A chest X-ray again demonstrated low lung volumes with some mild patchy opacities interpreted as likely atelectasis by the radiologist. These infiltrates resolved over the next two days as her oxygen saturation gradually improved.

She passed her SBT on POD 4. Her respiratory rate was 16–20 BPM with tidal volumes between 300 and 400 mL and an oxygen saturation of 97% on 40% FiO_2_ and PEEP of 5 cm H_2_O. She was able to generate an inspiratory force of −35 cm H_2_O and had a vital capacity >1 liter. The patient was extubated. Initially, the patient was able to breathe and speak comfortably. However, 12 hours later, she had a coughing episode during a position change that led to worsening tachypnea, agitation, and desaturation. She was returned to the upright position and an albuterol nebulizer was administered for audible “wheezing,” but her respiratory status did not improve. Even on bilevel positive airway pressure (BiPAP) assistance, she became progressively more agitated and confused and then somnolent and unresponsive. She was reintubated using a video laryngoscope. During intubation, no laryngeal edema or thick secretions were noted. Her oxygen saturation and ventilation quickly improved.

**Figure 1:**
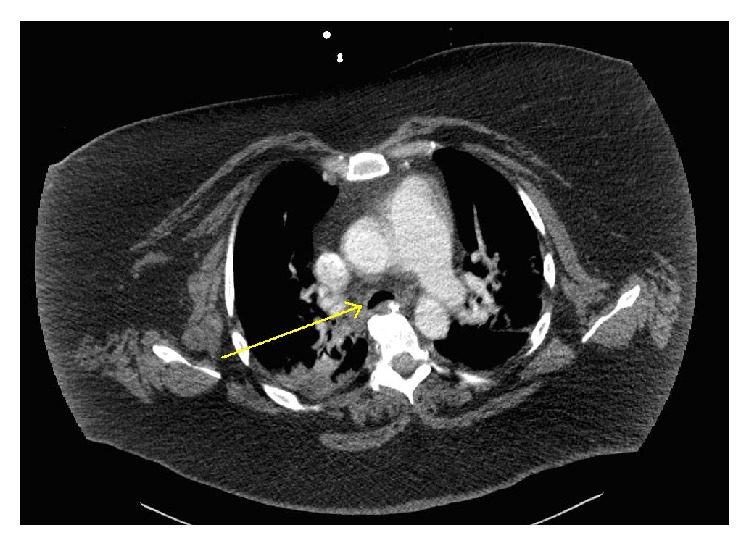


**Figure 2:**
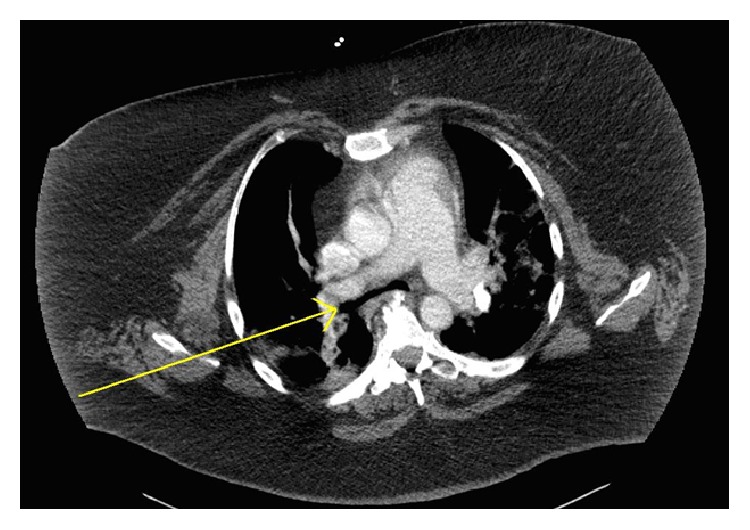


A CT scan found no evidence of pulmonary embolism; however, narrowing of distal trachea and main bronchi was noted (Figures 1 and 2). The radiologist's interpretation suggested tracheobronchomalacia. Unfortunately, the CT was not ordered to evaluate airway collapsibility; thus it was not timed for inspiration and exhalation. A normalizing white blood cell count, absence of fever, and lack of purulent tracheal secretions made pneumonia unlikely. Significant pulmonary edema was also unlikely, given a lack of radiologic findings and a lack of significant dependent edema.

BiPAP was immediately applied after a second extubation attempt on POD 7. However, despite favorable extubation criteria, the patient again developed violent coughing, stridor, and respiratory distress during a transient change to the supine position two hours after extubation. Racemic epinephrine and albuterol were administered, to no avail. She quickly became somnolent and then cyanotic. She was reintubated using a video laryngoscope. Again, the larynx was closely inspected, but no edema was seen.

**Figure 3:**
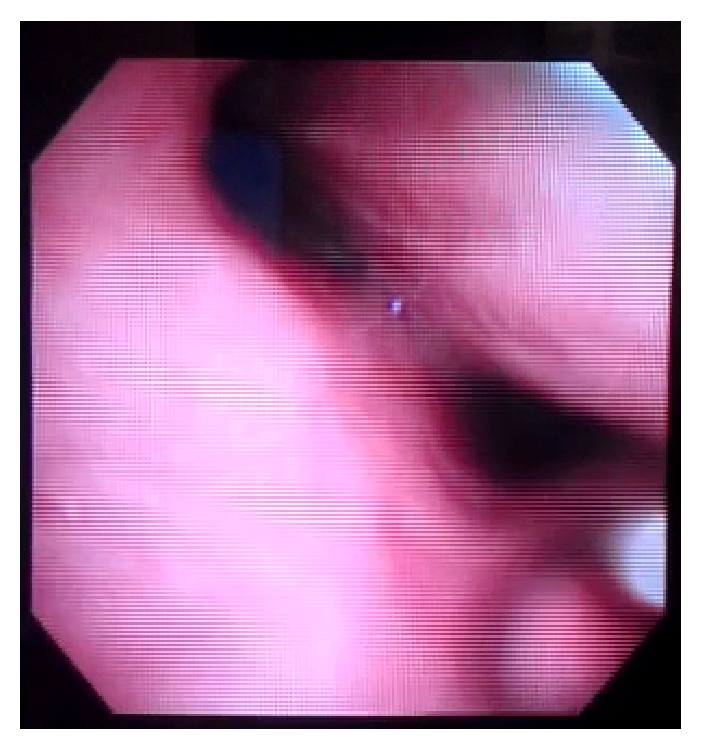
Inspiration.

**Figure 4:**
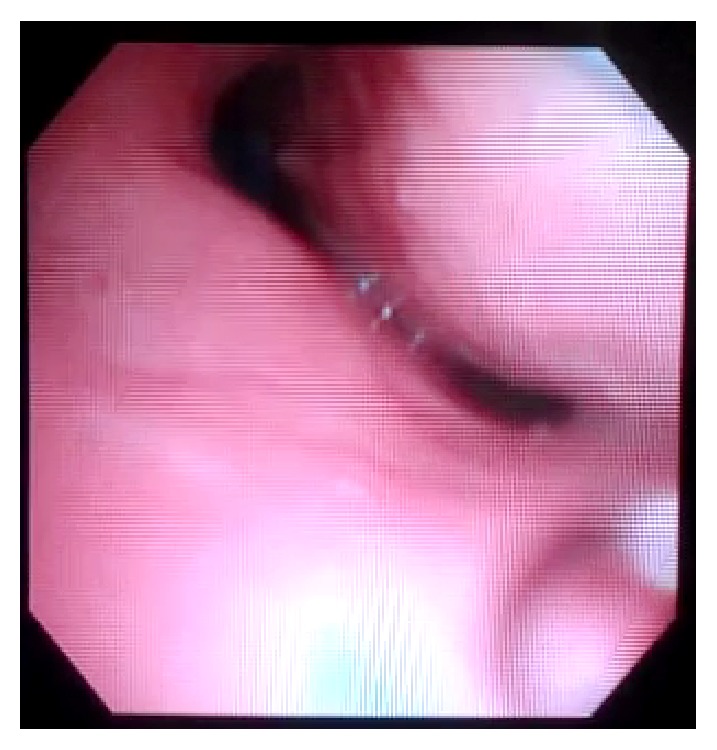
Exhalation.

**Figure 5:**
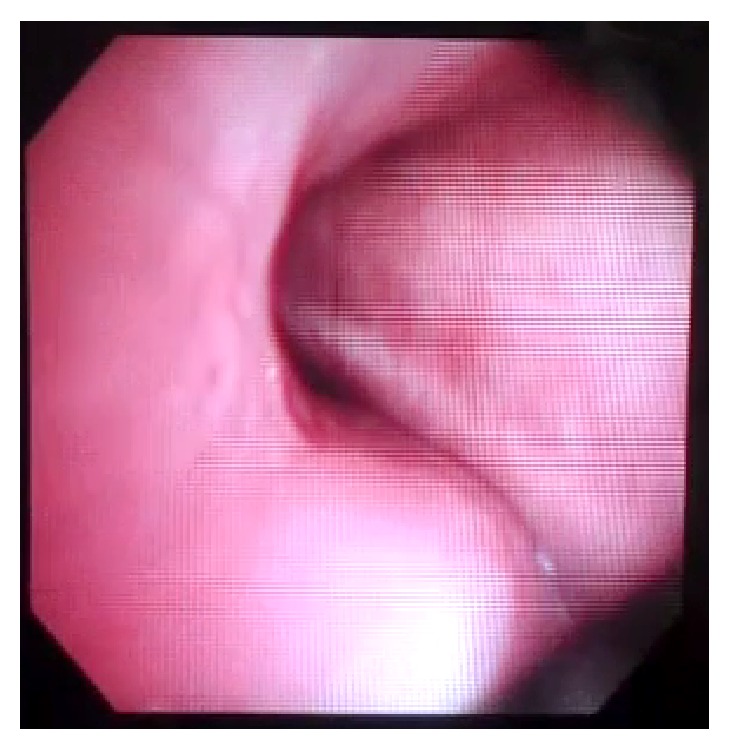
Cough.

A tracheostomy was performed on POD 9, and fiberoptic bronchoscopy was performed on POD 10 while the patient was breathing spontaneously. Dynamic airway collapse during exhalation was seen involving the trachea and the mainstem bronchi. The collapse was visually estimated to be greater than 50% of the airway diameter (Figures 3 and 4) during tidal respiration. During coughing, there was near total collapse of the trachea distal to the cuff of the tracheostomy tube (Figure 5). On POD 13 the patient was transitioned to tracheostomy mask trials and eventually discharged to a long term acute care hospital, 20 days after admission to the hospital.

## 3. Discussion

Some recent authors categorize pathologic dynamic airway collapse into two distinct forms that differ in pathogenesis, morphology, and histology [7, 8, 10]. TM results from pathologic softening of the cartilage in the airways. This can either lead to “flattening” of the trachea in the sagittal dimension, creating a crescent shape, or “narrowing” of the trachea in the coronal dimension which is sometimes referred to as a “saber sheath” trachea [8]. TM can be either a congenital or an acquired condition in most adult presentations. Acquired cases are often attributable to long-standing infection, COPD, trauma, tracheostomy, chronic tracheal inflammation (e.g., from polychondritis), external compression, or malignancy [1, 2, 4].

The second form of airway collapse results from anterior bowing of the posterior membranous portion of the trachea. Bowing is unrelated to any change in the rigidity of the cartilage and is better described as excessive dynamic airway collapse, since it is an exaggeration of the normal narrowing of the trachea during exhalation [11, 12]. In EDAC the posterior wall is thinner [10] and histologic evaluation of affected segments has shown changes in the elastic fibers of the pars membranacea [13]. Still, the terms TM and EDAC are often used interchangeably in current literature with ongoing debate about which is preferable [8, 9, 12, 14]. Thus we sometimes substitute a general term such as expiratory or dynamic airway collapse when reviewing the literature.

The finding of TM, from decreased cartilaginous rigidity, in this patient would be unexpected given the lack of risk factors. The clinical setting and the classical anterior bowing of the posterior membranous tracheal wall during bronchoscopy suggest EDAC as a more likely mechanism. Morbid obesity and the resulting increased intrathoracic pressure, especially in the supine position, would contribute to an exaggeration of normal expiratory airway collapsibility. Specific questioning of the patient and her parents did not reveal long-standing coughing or wheezing that would be consistent with preexisting EDAC masquerading as asthma or another respiratory problem. However, the patient did admit that over the preceding two years she had been sleeping in a recliner. In hindsight, this history could be suggestive of tracheal collapsibility.

The association between morbid obesity and EDAC has been demonstrated before in a population of COPD patients [15]. Although patients without COPD did not show a significant correlation between increased BMI and tracheal collapse, this patient's severely elevated BMI (62), increased secretions, airway reactivity, and inflammation from prolonged intubation may have played a similar role to COPD in this patient. The resulting increased work of breathing and increased flow velocities in the trachea during exhalation could exaggerate any existing dynamic flow obstruction. In support of this hypothesis, Kandaswamy et al. found a high prevalence of severe TM in ICU patients who required reintubation after passing a SBT or required an unexpectedly long ventilatory course. TM in this study was positively associated with morbid obesity [14].

This patient met extubation criteria in both instances of liberation from mechanical ventilatory support. She was fully awake and demonstrated adequate oxygenation, ventilation, and cough. Her rapid shallow breathing index was 53 to 66, suggesting a high probability that she could breathe without mechanical support [16]. Initially, after extubation, she appeared comfortable and was able to converse using complete sentences. This would make laryngeal edema, another common cause of extubation failure in female patients who have been intubated for several days, an unlikely diagnosis [17]. Prior to both reintubations, being placed in the supine position triggered a coughing episode and stridorous breathing. During reintubation the vocal cords were intentionally examined with the GlideScope (Verathon; Bothell, WA, USA) to rule out significant laryngeal edema, hematomas, or other causes of upper airway obstruction.

Unfortunately, making a conclusive diagnosis of TM/EDAC as the primary cause of respiratory failure is difficult. The clinical significance of tracheal collapse in a specific patient is difficult to quantify. Asymptomatic central airway collapse with exhalation may be a relatively common finding as suggested by a recent study of dynamic CT scans [3]. Furthermore, it has been shown that the degree of central airway collapse does not necessarily correlate with physiologic studies or baseline functional status [18]. Even so, this patient's sudden and severe respiratory failure heralded by stridor, coughing, dyspnea, and hypercapnia hours after what appeared to be a successful extubation fits well with the bronchoscopic findings and is supported by literature [4, 12, 14].

While a significant degree of airway collapse may have been tolerated by this patient preoperatively, a multitude of factors shifted the balance toward respiratory failure after surgery. In this patient, reduced FRC, respiratory muscle dysfunction from a high abdominal incision, atelectasis, edema, secretions, and tracheal reactivity from intubation contributed to an increased work of breathing. Unmasking of dynamic airway collapse has been described with anesthesia and progressive hypercapnic respiratory failure by other authors [4]. Affected patients may remain asymptomatic until stressed by a variety of factors that follow surgery or respiratory infection. While this patient remained intubated, positive airway pressure kept the airways expanded; however, when the positive pressure was removed with extubation, the patient developed respiratory distress and apparent stridor. The supine position and coughing or straining due to secretions further exacerbated the situation leading to worsening airway collapse from increased intrathoracic pressure.

Unfortunately, there are few treatment options for patients with TM and EDAC. Noninvasive positive pressure ventilation can be used in the short term to reduce expiratory airway collapse [1, 4, 12]. This was attempted by us as a rescue measure after the first extubation and immediately after the second extubation, without success. Surgery can rarely be an option. Tracheostomy alone may stent the collapsible portion of the proximal trachea [12]. This may have been the case in our patient, since she transitioned to spontaneous ventilation with a tracheostomy mask several days after the procedure. Tracheostomy could also potentially make airway collapse worse by eliminating the physiologic PEEP provided by a closed glottis. Also, tracheostomy may lead to TM later in life [4, 10]. Surgical stabilization of the posterior wall of the trachea can be performed in rare cases using either bone graft or mesh. This creates an external stent around the collapsible segment, although it is rarely done [4, 14]. More recently, silicone stents have been used to keep affected areas from collapsing [4]. Such stents, however, are prone to migration and impair ciliary secretion clearance.

In the present case we decided the best course of action was to make every effort to return our patient to her preoperative state through nutrition, diuresis of excess volume, reduction of atelectasis, and improved secretion clearance. Ultimately, a tracheostomy provided a safe way of securing the airway and allowing the patient to eventually transition out of the ICU. In addition to potentially stenting open an area prone to collapse, it reduced the overall work of breathing. The reduced respiratory effort may have relieved some of the increased intrathoracic pressure during exhalation and helped to reduce airway collapse during exhalation.

## 4. Conclusion

Excessive dynamic airway collapse (EDAC) may be a significant and underrecognized contributor to respiratory compromise in ICU patients who unexpectedly fail extubation. Traditionally, airway collapsibility has been categorized as TM which in adults is associated with long-standing smoking, infection, inflammation, trauma, or compression of the airways. These processes lead to softening of the cartilage. EDAC, however, exists as a distinct pathology that is unrelated to the above factors and may present unexpectedly in patients such as ours. Expiratory airway collapse is heralded by symptoms like refractory “wheezing,” coughing, stridor, and dyspnea which are often misinterpreted as asthma, COPD, or laryngeal edema.

These patients may not have clear symptoms at baseline; however, their airway collapse is worsened under conditions of respiratory stress. Processes that increase intrathoracic pressure or straining with exhalation such as morbid obesity, secretions, COPD, airway reactivity, and edema may exacerbate the baseline pathology leading to respiratory failure. Diagnosis is typically made through bronchoscopy, although newer modalities such as dynamic CT scanning are used ever more frequently. Treatment options include temporizing with positive pressure ventilation and supportive care until the respiratory stressors are corrected. Tracheostomy and in rare situations bronchial stents or surgery may be therapeutic options. Awareness of this disorder can facilitate earlier recognition and management.
